# Measurement Invariance of the Short Version of the Problematic Mobile Phone Use Questionnaire (PMPUQ-SV) across Eight Languages

**DOI:** 10.3390/ijerph15061213

**Published:** 2018-06-08

**Authors:** Olatz Lopez-Fernandez, Daria J. Kuss, Halley M. Pontes, Mark D. Griffiths, Christopher Dawes, Lucy V. Justice, Niko Männikkö, Maria Kääriäinen, Hans-Jürgen Rumpf, Anja Bischof, Ann-Kathrin Gässler, Lucia Romo, Laurence Kern, Yannick Morvan, Amélie Rousseau, Pierluigi Graziani, Zsolt Demetrovics, Orsolya Király, Adriano Schimmenti, Alessia Passanisi, Bernadeta Lelonek-Kuleta, Joanna Chwaszcz, Mariano Chóliz, Juan José Zacarés, Emilia Serra, Magali Dufour, Lucien Rochat, Daniele Zullino, Sophia Achab, Nils Inge Landrø, Eva Suryani, Julia M. Hormes, Javier Ponce Terashima, Joël Billieux

**Affiliations:** 1 International Gaming Research Unit, Psychology Department, Nottingham Trent University, Nottingham NG1 4FQ, UK; daria.kuss@ntu.ac.uk (D.J.K.); halleypontes@gmail.com (H.M.P.); mark.griffiths@ntu.ac.uk (M.D.G.); lpxcad@nottingham.ac.uk (C.D.); lucy.justice@ntu.ac.uk (L.V.J.); 2 Laboratory for Experimental Psychopathology, Psychological Sciences Research Institute, Université Catholique de Louvain, 1348 Louvain-la-Neuve, Belgium; joel.billieux@uni.lu; 3 Department of Social Services and Rehabilitation, Oulu University of Applied Sciences, 90220 Oulu, Finland; niko.mannikko@oamk.fi; 4 Research Unit of Nursing Science and Health Management, University of Oulu and Oulu University Hospital, 90014 Oulu, Finland; maria.kaariainen@oulu.fi; 5 Department for Psychiatry and Psychotherapy, University of Lübeck, 23538 Lübeck, Germany; Hans-Juergen.Rumpf@uksh.de (H.-J.R.); Anja.Bischof@uksh.de (A.B.); akgaessler@outlook.de (A.-K.G.); 6 EA 4430 Clinique Psychanalyse Développement (CLIPSYD), Université Paris Nanterre, France; U894 Centre de Psychiatrie et Neurosciences, (CPN), Inserm, 92000 Paris, France; romodesprez@gmail.com (L.R.); ymorvan@parisnanterre.fr (Y.M.); 7 EA 2931, Centre de Recherches sur le Sport et le Mouvement (CESRM), Université Paris Nanterre, 92000 Nanterre, France; laurence.kern@gmail.com; 8 Psychology Department, PSITEC EA 4074, Université Lille Nord de France, 59650 Villeneuve d’Ascq, France; amelie.rousseau@univ-lille3.fr; 9 LPS EA 849, Aix-Marseille University, 13007 Marseille, France; pierluigi.graziani@free.fr; 10 Psychologie, Langues, Lettres et Histoire Département, University of Nîmes, 30000 Nîmes, France; pierluigi.graziani@free.fr; 11 Institute of Psychology, ELTE Eötvös Loránd University, 1064 Budapest, Hungary; demetrovics@t-online.hu (Z.D.); orsolya.papay@gmail.com or kiraly.orsolya@ppk.elte.hu (O.K.); 12 Faculty of Human and Social Sciences, UKE—Kore University of Enna, Cittadella Universitaria, 94100 Enna, Italy; adriano.schimmenti@unikore.it (A.S.); alessia.passanisi@unikore.it (A.P.); 13 Department of Family Science and Social Work, Katolicki Uniwersytet Lubelski Jana Pawła II, 20-950 Lublin, Poland; bernadetalelonek@kul.lublin.pl; 14 Department of Psychology, Katolicki Uniwersytet Lubelski Jana Pawła II, 20-950 Lublin, Poland; chwaszcz@kul.pl; 15 Department of Basic Psychology, University of Valencia, 46010 Valencia, Spain; Mariano.Choliz@uv.es; 16 Department of Developmental and Educational Psychology, University of Valencia, 46010 Valencia, Spain; Juan.J.Zacares@uv.es (J.J.Z.); Emilia.Serra@uv.es (E.S.); 17 Service de Toxicomanie, Faculte de medicine Université de Sherbrooke, Longueuil, Qc, J4K 0A8, Canada; magali.dufour@usherbrooke.ca; 18 Department of Psychology and Educational Sciences, University of Geneva, 1205 Geneva, Switzerland; Lucien.Rochat@unige.ch; 19 Department of Psychiatry—Research Unit Addictive Disorders, University of Geneva, 1205 Geneva, Switzerland; Daniele.Zullino@hcuge.ch (D.Z.); Sophia.Achab@hcuge.ch (S.A.); 20 Department of Mental Health and Psychiatry—Addiction Division, University Hospitals of Geneva, 1205 Geneva, Switzerland; 21 Clinical Neuroscience Research Group, Department of Psychology, University of Oslo, 0317 Oslo, Norway; n.i.landro@psykologi.uio.no; 22 Department Psychiatry and Behavior, School of Medicine and Health Science, Atma Jaya Catholic University of Indonesia, Jakarta 14440, Indonesia; eva.suryani@atmajaya.ac.id or amyeva511@gmail.com; 23 Department of Psychology, University at Albany State University of New York, Albany, NY, USA; jhormes@albany.edu; 24 University Hospitals Cleveland Medical Center/Case Western Reserve University, Cleveland, OH 44106, USA; javier@incaas.org; 25 Addictive and Compulsive Behaviours Lab (ACB-lab), Institute for Health and Behaviour, University of Luxembourg, 4366 Esch-sur-Alzette, Luxembourg

**Keywords:** mobile phone use, smartphone use, Problematic Mobile Phone Use, Problematic Mobile Phone Use Questionnaire, psychometric testing, measurement invariance

## Abstract

The prevalence of mobile phone use across the world has increased greatly over the past two decades. Problematic Mobile Phone Use (PMPU) has been studied in relation to public health and comprises various behaviours, including dangerous, prohibited, and dependent use. These types of problematic mobile phone behaviours are typically assessed with the short version of the Problematic Mobile Phone Use Questionnaire (PMPUQ–SV). However, to date, no study has ever examined the degree to which the PMPU scale assesses the same construct across different languages. The aims of the present study were to (i) determine an optimal factor structure for the PMPUQ–SV among university populations using eight versions of the scale (i.e., French, German, Hungarian, English, Finnish, Italian, Polish, and Spanish); and (ii) simultaneously examine the measurement invariance (MI) of the PMPUQ–SV across all languages. The whole study sample comprised 3038 participants. Descriptive statistics, correlations, and Cronbach’s alpha coefficients were extracted from the demographic and PMPUQ-SV items. Individual and multigroup confirmatory factor analyses alongside MI analyses were conducted. Results showed a similar pattern of PMPU across the translated scales. A three-factor model of the PMPUQ-SV fitted the data well and presented with good psychometric properties. Six languages were validated independently, and five were compared via measurement invariance for future cross-cultural comparisons. The present paper contributes to the assessment of problematic mobile phone use because it is the first study to provide a cross-cultural psychometric analysis of the PMPUQ-SV.

## 1. Introduction

Mobile phones have become a ubiquitous technology and their use is widespread internationally. However, there appear to be differences in terms of technology use across various geographical regions according to the International Telecommunication Union (ITU). Recently, ITU Facts and Figures 2017 [1] demonstrated that mobile phone use has experienced the largest growth compared with other technologies over the last two decades. More specifically, worldwide mobile phone subscriptions per 100 inhabitants were 15.5 in 2001, 76.6 in 2010, and 103.5 in 2017. At the same time, subscriptions for landline telephones were 16.6 in 2001, 17.8 in 2010, and 13 in 2017. According to a study by ProQuest [2], the number of scientific papers and reports published on this topic has grown markedly. The study examined 26 scientific databases simultaneously (e.g., PsycINFO) using the search terms “mobile phone” or “cell* phone” and “smartphone”. It was reported that 490 academic outputs were published in 2001, 3225 in 2010, and 8224 in 2017 (these results referred to scholarly peer-reviewed journal articles, as well as trade journals, magazines, conference proceedings, and other reports).

Negative aspects related to mobile phone use are often conceptualised within the umbrella term of Problematic Mobile Phone Use (PMPU; [3,4]). According to Billieux and colleagues [4,5,6,7], PMPU can be understood as a heterogeneous and multidimensional construct involving the potential negative effects of mobile phone use. Accordingly, these authors formulated an integrative pathway model to account for the various types of problematic mobile phone use (i.e., dangerous, prohibited/antisocial, and dependent). Based on this model, each pathway to mobile phone overuse (i.e., extraversion pathway, reassurance-seeking pathway, impulsive pathway) is underlain by specific psychosocial factors and individual differences. Although maladaptive mobile phone use was initially considered a public health issue in child and adolescent populations [8,9,10,11], over the past decade, mobile phone use has been considered to involve potential risks for all populations across the different dimensions of problematic use, namely dangerous, prohibited, or dependent use [4].

Regarding general health issues traditionally associated with mobile phone use, several studies have shown significant associations between mobile phone use and users’ lifestyles and wellbeing. For example, Ezoe and colleagues [11] found that PMPU among Japanese female college students was associated with poor sleep, low physical activity, decreased work performance, and skipping breakfast. Similarly, Gallimberti and colleagues [12] observed that reading books, higher school marks, and longer hours of sleep were associated with low PMPU in Italian adolescents. Conversely, and in line with previous studies, other authors have reported PMPU to be positively associated with stress, depression, sleep disturbances, extraversion, female gender, young age, and poor academic or professional competence or performance [13,14,15,16,17,18,19,20,21,22]. Furthermore, Yang and colleagues [13] investigated the health and psychological problems associated with mobile phone use in adolescent Southern Taiwanese students and found that PMPU was associated with aggression, insomnia, smoking, suicidal tendencies, and low self-esteem.

For instance, two studies analysing young Swedish adults’ perceptions of the need of being available at all times via their mobile phones [14,15] reported that mobile phone use was positively associated with stress, depression, and sleep disorders. Similarly, a recent systematic review carried out by Elhai and colleagues [16] found that PMPU was usually related to depression, anxiety, chronic stress, and low self-esteem. However, only depression and anxiety were consistently related to this problematic use, with medium and small effect sizes, respectively. In another paper, the same authors even stated that while depression was inversely associated with social PMPU (e.g., social networking, messaging), anxiety was positively related to problematic use as a process or being consumption-based (e.g., news consumption, entertainment, relaxation) [17].

Associated behaviours, such as dependency and/or compulsiveness, have also been reported when individuals check their phone display, and even when not interacting with their mobile phone directly. This is because auditory and/or tactile notifications prompt thoughts that affect attention, and which negatively impact on performance [18] (a phenomenon coined as ‘technoference’; such use of mobile phones results in conflicts in interpersonal relationships and decreased wellbeing [19]). In addition to this, physical reactions, such as headaches and heat sensations, have been reported. In the same vein, Bickham and colleagues [20] found associations between PMPU and depression in North American adolescents. In sum, the existing evidence on smartphone use suggests a clear association between PMPU and decreased wellbeing, especially in young populations worldwide.

In relation to dangerous mobile phone use, PMPU has initially been negatively associated with safety behaviours [1,2], such as using mobile phones when driving, cycling, or walking. The importance of this factor is supported by the development of specific policies and regulations related to mobile phone use (i.e., to prevent road accidents). A study conducted in China [21] assessed unintentional injuries (i.e., road traffic injuries, pedestrian collisions, and falls) due to mobile phone use and psychopathological symptoms in adolescence. The most prevalent injury was collisions (followed by falls and other injuries), where adolescents experienced PMPU, as well as negative emotional, behavioural, and social adaptation symptoms. Another study from the United States (US) [22] reviewed the associations between motor vehicle crashes and PMPU in adolescents because drivers between 16 and 19 years in the US are the most likely to die as a consequence of distractions caused by mobile phones. The review evidenced that half of all adolescents texted on their mobile phone while driving.

Prohibition of mobile phone use (or its regulation) is another specific aspect of PMPU, and is usually associated with legal or public regulations. However, some individuals do not abstain from using phones in such circumstances (i.e., public spaces, such as libraries, cinemas, or theatres). According to Takao and colleagues [23], personality traits may be associated with these types of behaviours, such as self-monitoring (i.e., traits related to the tendency to control and regulate the public self) and approval motivations (i.e., the need for favourable evaluations from others). Both are associated with an extraverted personality, as indicated by previous research [14] because individuals with the extraversion trait are sensitive to social cues and peer pressure, which involves being prone to risk behaviours when using mobile phones constantly, even when their use is banned. This aspect of problematic mobile phone use can also be related to the fact that individuals use mobile phones in a way that interferes with social situations. A prototypical example is the act of snubbing someone in a social setting by using one’s mobile phone instead of interacting, a phenomenon referred to as “phubbing” [24,25].

The most studied type of negative outcome associated with mobile phone use is dependence, also conceptualised as a genuine addictive behaviour by some researchers [9,26]. The introduction of the internet and instant messaging (IM) on mobile phones (i.e., smartphones) has been associated with mobile phone dependence [21]. Moreover, it has also been associated with sociability levels of mobile phone users [27,28,29] and peer pressure [28]. However, studies examining peer pressure have reported slightly contradictory findings [29], where PMPU has not necessarily been associated with peer support or social acceptance. Therefore, it appears there is a potential association between mobile phone dependence (especially texting) and levels of sociability in adolescent and young adult populations [27,28,30]. Other factors usually associated with this type of problematic use include emotional symptoms (e.g., stress, anxiety, and depression [31,32,33]), reward seeking [26], and heightened impulsivity [2,26]. Moreover, specific mobile phone use patterns have also been associated with dependent use, except for some entertainment uses, such as downloading or playing mobile games [26,34,35], or using the mobile phone for travel bookings, online payments, and online shopping [34].

In sum, on the one hand, a few authors have claimed that the negative nature of dependent mobile phone use is not always severe, such as Chung [36], who argued that levels of dependence in South Korean female adolescent mobile phone users (i.e., withdrawal, maladjustment, tolerance, obsession, and flashiness) are associated with high levels of interpersonal solidarity (i.e., shared sentiments, intimacy, and similarities). Similarly, other scholars [37] have alerted researchers concerning the risk of overpathologizing everyday life behaviours in the context of behavioural addictions research, such as PMPU. On the other hand, Chóliz [38] has claimed that mobile phone addiction is a clinically relevant condition. Therefore, further research is warranted to assess the underlying motivations behind dependent use.

In relation to the cross-cultural assessment of PMPU, only a few studies have been conducted [39,40]. A number of different scales have been used [5,22,41,42], and according to a literature review by Pedrero and colleagues [42], the ‘gold standard’ scale is the Mobile Phone Problem Use Scale (MPPUS [3]). Unfortunately, the MPPUS is a unidimensional scale, which is problematic given the hypothesized multi-dimensional nature of PMPU. Moreover, the structural validity of the MPPUS was only tested with exploratory factor analysis (EFA), and needs to be confirmed in further studies using confirmatory factor analysis (CFA) and measurement invariance (MI). Another contemporary instrument to assess PMPU is the Problematic Mobile Phone Use Questionnaire (PMPUQ; [4]), which allows the measurement of the multi-dimensional nature of PMPU and was validated through the conjoint use of EFAs and CFAs. The scale assesses the three aforementioned specific types of PMPU. It was initially developed with a four-factor solution, but was recently reduced to a shorter version with three factors (dangerous use, prohibited use, and dependence) and updated to contemporary smartphone use (PMPUQ-SV; [33,35,43]). The fourth factor, related to the occurrence of financial problems, was removed due to the evolution of smartphones (i.e., smartphones being relatively cheap to use compared to when they were first introduced).

Subsequent studies—including some cross-cultural ones [33,35]—have evaluated the factor structure of the PMPUQ in its long or short versions via exploratory [35,43] and confirmatory [33,43,44] approaches in different populations (e.g., young adults [33,43], adults [35,43,44]), and different European languages, especially English [33,35,43,44]. However, psychometric results have been contradictory because some studies have reported adequate properties [33,35], while others have not [43,44]. Finally, to the best of the authors’ knowledge, no previous study has tested MI to establish the cross-validity of any of the PMPU scales (i.e., unidimensional or multidimensional) simultaneously across different languages using confirmatory approaches. This is a necessary step to move the field forward in order to establish cross-cultural MI of a scale to guarantee reliable and comparative findings across countries and languages.

The aim of the present study was to test the psychometric properties and measurement invariance of eight versions of the PMPUQ-SV. The languages selected were German, French, English, Finnish, Spanish, Italian, Polish, and Hungarian. A number of non-European countries using the same languages agreed to join the data collection in this first study. In addition to being able to perform future cross-cultural studies, there are a number of reasons for carrying out the present study to validate the PMPUQ-SV in several languages. Firstly, there is little empirical evidence regarding PMPU as a multidimensional construct, especially in adulthood. Secondly, PMPU has almost exclusively been investigated in relation to its addictive use rather than considering other potential problems (such as dangerous or prohibited use). Thirdly, the PMPUQ has been previously tested mostly using exploratory and confirmatory approaches, with no consistent results across different languages (e.g., English), but its MI across different languages remains to be investigated. Consequently, the present study investigated the multidimensional construct of PMPU across specific types of problematic mobile phone use described via the multi-group validation of the PMPUQ-SV across languages. Thus, the objectives were to (i) determine an optimal factor structure for the PMPUQ–SV among university populations using eight languages; and (ii) simultaneously examine the MI of the PMPUQ–SV across all languages in order to assess the linguistic comparability across the eight versions of the scale independently.

Therefore, the main purpose of the present study was to ascertain if the PMPUQ-SV is an appropriate psychometric tool for cross-cultural research. To the best of the authors’ knowledge, this is the first study to investigate the three-factor model in a multinational sample and the first to conduct MI on a multidimensional model of the PMPU across multiple linguistic scale versions. Thus, the present study will help fill an important gap in the field of PMPU and make a contribution to the research area because it comprises robust cross-cultural research examining mobile phone use and its associated problems.

## 2. Materials and Methods

### 2.1. Participants and Procedure

A total of 5209 respondents participated in the study, which builds upon the Tech Use Disorders (TUD; [45]) project. The items examined in the present study were part of a longer online survey including other questions (e.g., other scales concerning use of technology or personality traits). Participants were not forced to answer questions (because the survey was completely voluntarily). After cleaning the dataset (e.g., removing missing values), a sample of 3038 participants remained. The sample included adults engaged in higher education environments in 2015. The ethics committee of the Psychological Science Research Institute of the Université Catholique de Louvain (Belgium) approved the study protocol in 2014. Participants provided informed consent and voluntarily participated following an assurance of confidentiality and anonymity. The invitation to participate in the online survey (hosted on Qualtrics) used two recruitment strategies: (i) the present authors inviting undergraduates to participate via their respective universities during their 2015 and 2016 lectures; and (ii) via electronic invitations in academic online environments (e.g., university emails, university research participant pools, university social networks, and university virtual learning environments). Missing data were treated with pairwise deletion to maximise the statistical power, and cases were considered to be missing at random (MAR). This left a total sample size of 3038 participants with some not included for several reasons (e.g., young participants were not yet drivers, etc.). The sample breakdown by each respective language is shown in Table 1, alongside key socio-demographic data and reliability estimations.

Consequently, a total of eight languages were included in the present study (see Table 1), which were provided by 14 countries participating in the present study via their respective academic environments: German (i.e., Germany: 12.61% of the sample), French (i.e., Belgium: 16.06%; France: 10.60%; Switzerland: 3.39%; Canada: 5.13%; others who filled in the French adaptation chose not to report their country: 0.23%), English (i.e., United Kingdom (UK): 1.81%, Norway: 1.71%; US: 0.13%; Indonesia: 0.20%), Finnish (i.e., Finland: 14.78%), Spanish (i.e., Spain: 5.13%), Italian (i.e., Italy: 9.48%), Polish (i.e., Poland: 8.49%), and Hungarian (Hungary: 10.24%).

### 2.2. Instrument

To assess potential PMPU, the 15-item PMPUQ-SV [33,35,43] was adapted from English into the other seven languages using a standard translation and back-translation method [46], except for French (as it was the original language [4]) (see Appendix A). Each subscale comprised five items, which were scored from 1 (‘I strongly agree’) to 4 (‘I strongly disagree’), except for the items that were reverse scored [35] (see Table 1 for descriptive item scores). Overall scores ranged from 15 to 60, with higher scores indicating more potential problems due to mobile phone use. The Cronbach’s alphas of the PMPUQ-SV across all languages ranged from 0.56 (English version; prohibited use) to 0.90 in the present study (German version: dependence; French version: dependence; English version: dangerous use).

### 2.3. Analysis

#### 2.3.1. Understanding Measurement Invariance

To investigate whether the PMPUQ-SV is psychometrically valid for use across different languages, an analysis of MI was conducted using multigroup confirmatory factor analysis (MGCFA). MI establishes whether various aspects of the latent structure of a model remain stable across multiple groups, being run in an iterative manner with a set of increasingly constrained confirmatory factor analyses (CFAs). Comparison tests are then undertaken to determine if reliable differences exist between these models [47], which would suggest groups have reliable variations at those specific levels. The first step in this procedure was to conduct individual CFAs in each language group and investigate model fit. Following this, a set of constrained and planned models were implemented as follows. Constraints are given in square brackets and are cumulative throughout:

- A test of configural (or ‘pattern’, (groups)) invariance that investigates whether the same number of factors and their respective items are the same across groups (i.e., does the specified CFA structure replicate across the groups tested?). Support for configural invariance would suggest that the three-factor solution of the PMPUQ-SV and respective items per factor are valid across groups.
- A test of metric (or ‘weak’, (loadings)) invariance that estimates whether the factor loading strengths are equivalent across groups. Metric invariance suggests that participants understand and respond to items in the same way across groups.
- A test of scalar invariance (or strong invariance, (thresholds)) investigates if group differences in factor means are unbiased [48], meaning latent scores can be compared across groups.
- A test of strict invariance (residuals) estimates whether observed items have the same residuals, meaning that items have the same measurement error terms across groups.
- An additional fifth model of strict invariance and equally constrained (means) tests if the entire mean structure is invariant. If supported, this suggests that the means of both the latent variables and observed variables are invariant across groups.

#### 2.3.2. Ordinal Data Analysis

Given that all PMPUQ-SV items are assessed on ordinal scales, models that could support non-continuous item analyses were employed [49]. Therefore, multigroup analyses were run using the R program with RStudio [50] using the Lavaan [51], Psych [52], and SemTools [53] packages, all of which have options for assessing ordinal data in a CFA framework (see [54] for a tutorial). Accordingly, thresholds rather than intercepts were constrained. In all CFA models, correlation matrices used were polychoric and model fit statistics were estimated using diagonally weighted least squares scale-shifted (DWLSSS). DWLSSS has been found to be a more effective estimation method for ordinal data than maximum likelihood [55,56], the default estimation for most statistical software.

## 3. Results

### 3.1. Factor Structures

As the PMPUQ-SV is a relatively new scale and its first aim was to assess its psychometric properties to ascertain potential reasons concerning previous contradictory results [33,35,43,44], cut-off points for all indices were taken prudently. Firstly, individual CFAs were performed for the overall sample and individually in each language. The correlation matrix and factor loadings of PMPUQ-SV items across all linguistic versions can be found in Table 2 and Table 3, respectively. Cut-off values for fit indices were applied as follows (although caution must be taken as these cut-off values appear to result in lower Type II error rates (with acceptable costs of Type-I error rates [57,58,59,60,61])): a Comparative Fit Index (CFI) between 0.90 and 0.95 is indicative of acceptable fit relative to the independent model, and from 0.95 is considered a good fit; Tucker Lewis Index (TLI) values greater than 0.90 have been used as acceptable fit models in the past, but since 2000, this has been increased to approximately 0.95 indicating good fit; Root Mean Square Error of Approximation (RMSEA) values less than or equal to 0.05 can be considered as good fit, values between 0.05 and 0.08 are acceptable, and greater than 0.08 could be considered a mediocre fit, but higher than 0.10 are considered a poor fit (ideally, if the RMSEA is greater than 0.05, the fit of the model is ‘close’ (i.e., such a model has a specification error, but this is not large; then sample size is a critical factor)); Standardised Root Mean Square Residual (SRMR) values must be less than 0.08 or close to 0.09 (or 0.10), as it is the most sensitive index to models with misspecified factor loadings, and a combination rule has been suggested (i.e., if the RMSEA is greater than 0.05 or close to 0.06, then the SRMR should be greater than 0.06, or close to 0.09–0.10, as it is usually acceptable for sample sizes that are equal to or less than 250 [59]). However, although reported here for the sake of transparency, χ^2^ was not used to assess model fit, as it has been found to artificially inflate with an increasing sample size [62].

As can be seen in Table 4, the PMPUQ-SV in German, French, and English yielded good model fit statistics, whilst Hungarian and Finnish versions yielded adequate model fit statistics using less conservative cut-off scores. The Spanish version was almost acceptable, while the Italian and Polish versions had poor fit and were thus not carried forward for MI testing. Including the Italian and Polish versions would likely lead to the multigroup analysis immediately failing because the models already differ between the validated and non-validated languages in terms of factor numbers or structure. The English version was also removed because the highest response score for Item 12 (public transport) was not endorsed by any participants (with Lavaan requiring at least one response per level as a prerequisite).

To test for MI across languages, a series of MGCFAs with increasing constraints were conducted. The degree of difference (Δ) between the pairs of nested models was assessed using ΔCFI, ΔRMSEA, and ΔSRMR, as recommended by Chen [63], with respective cut-off values of ≤0.01, ≤0.015, and ≤0.03 for metric invariance and ≤0.01 for scalar invariance [64]. Satorra-Bentler χ^2^ difference tests were also calculated between the nested models, although again, these have been found to produce unreliable estimates for large sample sizes, and therefore need to be interpreted with caution. Successive models were only calculated if the previous less constrained invariance in the hierarchy was at least partially supported.

Configural invariance was supported because the majority of fit indices were adequate, and so the next levels of constraint were investigated. Satorra-Bentler tests between all subsequent MI models were reliably different (*p* < 0.001; see Table 5), suggesting that each successive model had a poorer fit than the previous one (this provides evidence against invariance). At each stage, the changes in fit indices were inspected to assess whether this conclusion could be supported. Across all models, the changes in all fit indices were well below the pre-specified cut-off Δ-values, with the exception of ΔCFI for the scalar, strict, and mean models, which exceeded the ≤0.01 threshold at 0.012, 0.014, and 0.012, respectively. Considering the excellent values for the remaining fit indices at each stage, overall, the MI results provide evidence for metric invariance and partial evidence for scalar, strict, and mean invariance.

### 3.2. Response Rates

Finally, in order to explore potential reasons for the violated ΔCFI for the fifth (means) model and if any items could be flagged up for refinement in future iterations, the response rates for each item were investigated across all different linguistic versions. As can be seen in Table 6, across all items for all languages, 75.2% of responses were in the ‘disagree’ or ‘strongly disagree’ categories. However, as items were reversed (i.e., 2, 4, 8, 9, 10, 11, 13, and 14), it should be interpreted that, for example, strongly agreeing on Item 14 (i.e., ‘I use my mobile phone while driving, even in situations that require a lot of concentration’) is more indicative of PMPU (i.e., dangerous use), but strongly agreeing on Item 12 (i.e., ‘When using my mobile phone on public transport, I try not to talk too loud’) is less indicative of PMPU (i.e., prohibited use). Moreover, a pair of items (i.e., Item 12 and Item 14) showed a particularly skewed response pattern with less than 7.6% and 4.9%, respectively, of respondents endorsing PMPU, to some degree, with both statements. Response patterns such as these may suggest that participants were not able to identify with these behaviours and that particular items that comprise the PMPUQ-SV may not be able to adequately discriminate respondents into distinct groups (i.e., as the vast majority of respondents reject these items). Taken together, the MI results illustrate that some of the items of the PMPUQ-SV with more skewed response patterns may prove consistently difficult to identify with across French, German, Hungarian, Finnish, and Spanish respondents.

## 4. Discussion

The objectives of the present study were to determine an optimal factor structure for the PMPUQ–SV among university populations using eight different language versions, and to examine the MI of the PMPUQ–SV across all linguistic versions and across the eight versions. Taken together, the findings suggest that the PMPUQ-SV is a potentially appropriate psychometric tool to screen for prohibited, dangerous, and dependent mobile phone use in adults from countries using these languages (e.g., Europe and America). However, its psychometric properties can be nuanced depending on the respective language. Despite this, there are several potential reasons for adopting this tool. First, it is a very good psychometric tool for French and German mobile phone users, as its fit indices showed good fit in comparison with the other languages (i.e., CFI_French and German_ = 0.97; TLI_French and German_ = 0.97; RMSEA_French and German_ = 0.07; SRMR_French_ = 0.07, SRMR_German_ = 0.08). Second, in psychometric terms, the English and Hungarian versions were considered robust and the Finnish and Spanish versions were considered acceptable. However, the Italian and Polish versions did not meet the psychometric requirements.

### 4.1. Main Findings

Using the overall sample, the PMPUQ-SV performed well across its three theoretical factors in terms of their internal consistency, factor loadings, and CFA results. Only two elements were close to the limit of being psychometrically acceptable [59]. More specifically, the Cronbach’s alpha for prohibited use was low because it is a coefficient sensitive to the number of items (i.e., the subscale only had five items), and it demonstrated that there was no poor interrelatedness between items or heterogeneous constructs, except for Item 6 (i.e., ‘*I don’t use my mobile phone in a library*’) relating to prohibited use. This item does not load strongly enough and could perhaps be dropped in future PMPUQ versions. Furthermore, as evidence for metric invariance demonstrated, factor loadings were similar across the MI groups, so Item 6 was poor across countries. In fact, reliabilities were quite poor for prohibited mobile phone use [65].

It was also demonstrated that the RMSEA value was not ideal [59,60]. The French and German versions were psychometrically excellent, although the latter had the same issue with an acceptable reliability for prohibited use. Consequently, more in-depth research is needed to explore the phenomenology of this specific aspect of PMPU. Similarly, the English and Hungarian versions fitted the model well, although the reliability of the English version using Cronbach’s alpha was only acceptable [66]. In addition, the Finnish and Spanish versions can be argued to be acceptable with restrictions due to their mediocre TLI, RMSEA, and SRMS values.

In sum, half of the linguistic versions tested in the present study fitted the proposed model well (i.e., ordered by their respective goodness of fit: French, German, Hungarian, and English). However, two require future testing (i.e., Finnish and Spanish) and the other two (i.e., Polish and Italian) require further review in relation to potentially different mobile phone use patterns in some countries and/or to methodological aspects (e.g., the translation and back-translation method applied), because both had large enough sample sizes to test its factor structure using a confirmatory approach. Regarding the English version, previous studies using this tool have also shown other psychometric weaknesses in relation to its reliability (e.g., in other studies, the α for prohibited use was 0.59 [35], and the α for the dangerous subscale was 0.67 [35], or 0.42 [43]), which are in line with the present findings regarding the limited internal consistency for prohibited use. Consequently, this needs to be cautiously interpreted due to the short length of this particular subscale. However, in relation to previous studies using the full PMPUQ or its short version, its factor structure usually corroborated the underlying theoretical model, except for the English studies, which reported a two-factor solution [43,44].

The lower loadings achieved in the overall sample for one of the items on the dangerous use subscale (i.e., *‘I try to avoid using my mobile phone when driving on the motorway’*), and four items on the prohibited use subscale (e.g., *‘When using my mobile phone on public transport, I try not to talk too loud’*), could be due to two reasons. First, mobile phone users are probably in a more pre-contemplative stage (i.e., they may not consider their mobile phone behaviour as problematic when asked about it, possibly denying or resisting this possibility), which is in line with research on compulsive internet use and other addictive behaviours [67,68]. Second, some items may not have been appropriate in the present day and age. For instance, *‘I don’t use my mobile phone in a library’*, which appeared appropriate in the present study because university samples were used, but (i) not all respondents may use libraries given the ease in which reading materials can be accessed remotely; and (ii) those who are library users can access their mobile phones using silent option modes (e.g., for checking the time or *Facebook* notifications, using IM, navigating, or listening to music through headphones).

When looking at the descriptive findings, the study also demonstrated that there appeared to be common usage patterns and preferences in relation to mobile phones for dangerous and prohibited use in the respective language versions. Items related to dangerous and prohibited use were very extreme (either strongly agree or disagree on the Likert scales), whereas items related to dependent use were more evenly spread across the response categories (from strongly agree to strongly disagree on the Likert scales). This may explain why a lower internal consistency was found for prohibited use, as well as the fact that the subscale only had five items. Underlying cultural differences may also explain this (e.g., English-speaking participants may have the most diversity in response to Item 12 (the item with lowest loadings on the prohibited use factor); for example, some cultures do not appear to mind talking on a bus, whereas other cultures do not like it at all). The study also demonstrated, in reference to the response patterns in Table 6, that participants endorsed much fewer PMPU behaviors when responding to items on the dangerous *(M* = 5.025) and prohibited factors (*M* = 5.9), relative to the dependency factor (*M* = 12.24). Furthermore, in a previous study [43], Item 12 was excluded from the analysis, because it did not share variance in the body of items.

The results in the present study also demonstrated that cultural differences in self-reported mobile phone usage patterns and contextual factors must be investigated in greater depth (e.g., driving regulations in the countries where the study was conducted). The PMPUQ-SV may be a good tool to initially screen for potential PMPU among adult mobile phone users in some languages (French, German, Hungarian, English, Finnish, and Spanish) if tested independently per country. However, the scale is only useful for mobile phone users who drive vehicles. Consequently, dangerous use is only associated with driving behaviour, instead of other dangers when using mobile phones (e.g., crossing the road). In future developments of the PMPU, it is recommended that the construct should not only be assessed with items related to driving behaviour [3,4,25] because other dangerous behaviours also exist and have been reported in recent research regarding safety, which can be included in future iterations (e.g., collisions or injuries when cycling or walking [24], such as ‘*I use my mobile phone whilst crossing the road*’ [43]), especially if new scales are going to be tested using children, adolescents, or adults that are not drivers. Conversely, in countries like the US where driving is permitted during mid-adolescence [25], items addressing driving behaviours when using mobile phones are recommended (e.g., PMPUQ-SV). In other words, while almost all European countries allow driving individuals to drive from the age of 17 or 18 years old, in other countries, the ages at which individuals can drive are lower, such as 14–16 years old.

Regarding the strongest MI results, only the French, German, Hungarian, Finnish, and Spanish results can be compared when using these versions in a cross-cultural study [68] because it was only in these countries that configural and metric invariances resulted in obtaining the expected value [63,64]. However, the other types of invariance (i.e., scalar, strict, and mean models) slightly exceeded the threshold, but the model can still be considered tenable. In sum, the present findings suggest that the factor structure, loadings and intercepts, and residuals of the PMPUQ-SV are invariant across the French, German, Hungarian, Finnish, and Spanish language versions. Therefore, the present study provides evidence for the equality of meaning of the problematic mobile phone use construct in five out of eight languages, further providing confidence in future use of the PMPUQ-SV in cross-cultural research on PMPU. For instance, among some of the countries where the TUD project [45] was developed (i.e., Belgium, France, Switzerland, Canada, Germany, Hungary, Finland, and Spain), it appears that cross-cultural data can be reliably compared.

### 4.2. Limitations

The main potential limitations of the present study are the sampling and characteristics of the participants (i.e., convenience community-based self-selected samples), who were adults studying or working in universities (or who were related to those who study or work in higher education institutions). Nevertheless, a large sample was collected during the same period (i.e., in 2015), using similar strategies, and the same online survey, in order to guarantee the standardization of the procedures for collecting reliable data from the three specific aspects of PMPU. The data were also self-reported and are therefore subject to well-known biases and limitations that are inherent within such a methodology. The purpose of the present study was to evaluate the cross-cultural robustness of the PMPUQ-SV to facilitate the development of future epidemiological studies across different cultures as these studies can help better ascertain the potential problems on the phenomenology of maladaptive mobile phone use from a psycho-sociological perspective. The PMPUQ-SV is appropriate for specific types of mobile phone users and has partially been cross-validated, but still presents some weaknesses which need to be studied in future research (i.e., reliability and language adaptability).

### 4.3. Future Research Directions

PMPU is still open to debate in relation to its potential health and educational harms in individuals’ daily lives. For instance, it is not clear if PMPU results from a contemporary psychosocial problem (facilitated through this technology and the online behaviours associated with it in individuals’ daily lives) or from other potentially addictive technological behaviours [37]). PMPU is situated somewhere on the continuum between the absence of problems to severe problems, ranging from a normal daily behaviour to potentially dysfunctional behaviours (or as the consequence of an existing disorder [38]). Furthermore, mobile phones are being increasingly used by adolescents and young adults worldwide [1], and given that they are usually utilized mainly for communicative purposes (i.e., information and maintenance of social relationships), some degree of constant use is expected in Eastern and Western societies.

In recent years, research on PMPU has bloomed in East Asian countries, where the condition is often viewed and classified as an addictive behaviour. Recent studies conducted in this region have indicated the moderating and mediating roles of several sociodemographic factors (e.g., gender), usage patterns (e.g., history of mobile phone use), and psychological variables (e.g., personality traits, emotion regulation skills) [69,70,71,72,73]. Most of this research focused on addictive usage patterns (with scales such as the Mobile Phone Addiction Index [74]), and it would thus be relevant to adapt the PMPUQ-SV to these contexts to provide a tool able to measure different types of PMPU. Such adaptation would also allow for interesting cross-cultural studies to be conducted between, for example, Asian and European countries.

One of the first literature reviews that examined both problematic internet and mobile phone use between 1991 and 2005 using five scientific databases determined, at that time, that mobile phone addiction symptoms were less consistently reported than internet addiction symptoms [75]. Sanchez-Carbonell and colleagues stated in 2008 that the use of synchronous apps (such as chatting apps and online games) might increase the likelihood of developing an addictive behaviour, due to the time lapse between engaging in the act and receiving a reward. A recent longitudinal study [30] partially confirmed this hypothesis (especially in relation to social networking apps and messaging services such as *WhatsApp* and *Facebook*). However, recent research on PMPU has not provided this evidence yet [35], in comparison with other internet-related problems, such as gaming, which appears to be the most prevalent because of the immediate rewards [76]. A recent review of cell-phone addiction [77] concluded (irrespective of whether or not it is a genuine addiction) that mobile phones give rise to problems that increasingly affect daily life. For instance, even with the risk of unlimited use (due to the affordability of contracts), the conceptualisation of this problematic mobile phone behaviour is still debated. In general, there is still an overlap in definitions of problematic behaviours related to online activities, including PMPU, Internet addiction, gaming disorder, social network use disorder, and others. Future studies are needed to gather evidence on how a nomenclature can progress and improve.

## 5. Conclusions

The present study is the first to ascertain that the PMPUQ-SV is an appropriate psychometric tool for cross-cultural comparisons to determine future prevalence estimates of multidimensional PMPU (i.e., dangerous, prohibited, and dependent mobile phone use). This is the first study that has investigated PMPU in an international sample, conducting MGCFAs and MI on a multidimensional model of the PMPUQ-SV across multiple language versions. An optimal factor structure (i.e., three-factor model) was found for the PMPUQ–SV among different university populations using six language versions (French, German, Hungarian, English, Finnish, and Spanish), and the MI of the PMPUQ–SV was examined across eight linguistic versions. The results indicate that five of the language variants (i.e., French, German, Finnish, Spanish, and Hungarian) are comparable for future cross-cultural studies. The PMPUQ-SV has been validated for almost all languages tested in order to be used independently in countries using these languages, and parts of these versions can be used for cross-cultural comparisons. The present study contributes to the behavioural addictions field by cross-validating results that can be used for future cross-cultural research on PMPU.

## Figures and Tables

**Table 1:** Demographic Information and item scores across all eight adaptations of the PMPUQ-SV.

	All	German	French	English	Finnish	Spanish	Italian	Polish	Hungarian
*N*	3038	383	1076	117	449	156	288	258	311
Women (*N* (%))	2193 (72%)	262 (68%)	829 (77%)	89 (76%)	308 (69%)	123 (79%)	190 (66%)	187 (72%)	205 (66%)
Age in (Yrs; mean (*SD*))	26.505 (9.395)	25.204 (6.602)	25.22 (10.034)	27.735 (11.319)	28.296 (9.06)	28.045 (11.642)	28.576 (9.555)	25.279 (6.965)	27.833 (9.032)
PMPUQ Score (mean (*SD*))	27.156 (6.869)	26.976 (6.547)	27.005 (7.177)	28.145 (5.564)	26.938 (6.584)	28.5 (6.696)	29.59 (6.533)	28.961 (6.399)	23.539 (6.159)
**Cronbach’s α**									
*Dangerous use*	0.84	0.88	0.81	0.90	0.87	0.86	0.86	0.77	0.89
*Prohibited use*	0.69	0.65	0.74	0.56	0.62	0.66	0.68	0.66	0.75
*Dependent use*	0.88	0.90	0.90	0.83	0.85	0.85	0.84	0.82	0.89
**Item Scores (mean (*SD*))**									
1. Easy not to use mobile	2.359 (1.034)	2.460 (0.991)	2.352 (1.095)	2.462 (0.915)	2.341 (1.030)	2.455 (0.905)	2.581 (0.921)	2.391 (1.009)	1.971 (0.991)
2. Use mobile when driving ^R^	1.569 (0.869)	1.525 (0.789)	1.402 (0.784)	1.393 (0.754)	1.933 (0.968)	1.549 (0.930)	1.874 (0.904)	1.721 (0.917)	1.354 (0.773)
3. Don’t use when forbidden	1.737 (0.943)	1.593 (0.866)	1.830 (0.981)	1.632 (0.826)	1.506 (0.869)	1.821 (0.962)	1.839 (0.936)	2.128 (1.003)	1.508 (0.823)
4. Difficult not to use mobile ^R^	2.179 (0.965)	2.285 (0.892)	2.214 (1.039)	2.308 (0.825)	2.049 (0.941)	2.393 (0.884)	2.345 (0.889)	2.236 (0.926)	1.772 (0.867)
5. Avoid using on motorway	1.417 (0.866)	1.324 (0.752)	1.467 (0.931)	1.308 (0.760)	1.379 (0.755)	1.319 (0.825)	1.360 (0.751)	1.721 (1.123)	1.296 (0.755)
6. Using mobile in library	2.354 (1.037)	2.590 (0.988)	2.423 (1.086)	2.624 (0.953)	2.336 (0.998)	2.757 (0.918)	2.205 (0.986)	2.031 (0.994)	1.965 (0.927)
7. Easy to live without mobile	2.473 (0.995)	2.527 (0.900)	2.377 (1.052)	2.778 (0.862)	2.715 (0.993)	2.653 (0.855)	2.784 (0.854)	2.442 (0.937)	1.929 (0.902)
8. Dangerous situations ^R^	1.721 (0.965)	1.574 (0.782)	1.828 (1.102)	1.410 (0.697)	1.686 (0.846)	1.730 (0.996)	1.839 (0.929)	2.070 (1.011)	1.302 (0.636)
9. Use mobile when forbidden ^R^	1.581 (0.806)	1.655 (0.753)	1.440 (0.754)	1.701 (0.757)	1.579 (0.804)	1.822 (0.900)	1.909 (0.954)	1.694 (0.829)	1.431 (0.701)
10. Lost without mobile ^R^	2.205 (0.986)	2.016 (0.859)	2.213 (1.056)	2.607 (0.909)	2.245 (0.953)	2.160 (0.980)	2.345 (0.932)	2.395 (0.945)	1.939 (0.930)
11. Driving danger mobile ^R^	1.379 (0.762)	1.295 (0.600)	1.437 (0.886)	1.248 (0.642)	1.425 (0.738)	1.299 (0.638)	1.529 (0.827)	1.434 (0.715)	1.125 (0.448)
12. Public transport	1.462 (0.711)	1.496 (0.647)	1.369 (0.709)	1.530 (0.566)	1.410 (0.751)	1.542 (0.668)	1.586 (0.684)	1.659 (0.869)	1.476 (0.621)
13. Hard to turn off mobile ^R^	1.999 (0.980)	1.984 (0.880)	1.911 (1.023)	2.325 (0.936)	1.795 (0.920)	2.278 (0.978)	2.399 (0.948)	2.019 (0.906)	1.994 (0.987)
14. Driving concentration ^R^	1.247 (0.597)	1.188 (0.459)	1.171 (0.505)	1.231 (0.578)	1.332 (0.703)	1.306 (0.651)	1.482 (0.787)	1.415 (0.755)	1.087 (0.353)
15. Use mobile in silent place	1.496 (0.749)	1.457 (0.620)	1.578 (0.851)	1.590 (0.697)	1.287 (0.594)	1.558 (0.769)	1.538 (0.672)	1.605 (0.813)	1.373 (0.659)

Note: ^R^ = reverse coded; PMPUQ-SV: Problematic mobile phone use questionnaire short version.

**Table 2:** Correlation matrix of the 15 PMPUQ-SV items across all language adaptations.

	Item Number														
	***1***	***2***	***3***	***4***	***5***	***6***	***7***	***8***	***9***	***10***	***11***	***12***	***13***	***14***	***15***
***1***	***-***														
**2**	0.19 ***	-													
**3**	0.25 ***	0.22 ***	-												
**4**	0.58 ***	0.19 ***	0.30 ***	-											
**5**	0.09 ***	0.51 ***	0.33 ***	0.05 ***	-										
**6**	0.36 ***	0.07 ***	0.33 ***	0.33 ***	0.14 ***	-									
**7**	0.78 ***	0.22 ***	0.28 ***	0.56 ***	0.11 ***	0.39 ***	-								
**8**	0.19 ***	0.45 ***	0.31 ***	0.32 ***	0.35 ***	0.14 ***	0.20 ***	-							
**9**	0.23 ***	0.44 ***	0.51 ***	0.42 ***	0.31 ***	0.29 ***	0.29 ***	0.52 ***	-						
**10**	0.56 ***	0.13 ***	0.22 ***	0.56 ***	0.02	0.25 ***	0.63 ***	0.25 ***	0.32 ***	-					
**11**	0.12 ***	0.57 ***	0.24 ***	0.22 ***	0.41 ***	0.02	0.13 ***	0.53 ***	0.40 ***	0.19 ***	-				
**12**	0.08 ***	0.06 ***	0.18 ***	0.04 *	0.19 ***	0.12 ***	0.10 ***	0.08 ***	0.12 ***	0.01	0.08 ***	-			
**13**	0.53 ***	0.15 ***	0.27 ***	0.56 ***	0.05 *	0.23 ***	0.54 ***	0.25 ***	0.37 ***	0.62 ***	0.20 ***	0.09 ***	-		
**14**	0.15 ***	0.67 ***	0.32 ***	0.23 ***	0.49 ***	0.06 *	0.18 ***	0.56 ***	0.53 ***	0.20 ***	0.68 ***	0.20 ***	0.30 ***	-	
**15**	0.25 ***	0.08 ***	0.41 ***	0.25 ***	0.22 ***	0.41 ***	0.28 ***	0.22 ***	0.33 ***	0.20 ***	0.15 ***	0.38 ***	0.27 ***	0.21 ***	-

Note: All correlations are polychoric and *p*-values are FDR corrected for multiple comparisons, * *p* < 0.05, *** *p* < 0.001; PMPUQ-SV: Problematic mobile phone use questionnaire short version.

**Table 3:** Factor loadings of the PMPUQ-SV items across all languages.

	Factor Loadings		
	Dangerous Use	Prohibited Use	Dependent Use
14. Driving concentration	0.88		
11. Driving danger mobile	0.74		
2. Use mobile when driving	0.73		
8. Dangerous situations	0.73		
5. Avoid using on motorway	0.57		
9. Use mobile when forbidden		0.80	
3. Don’t use when forbidden		0.63	
15. Use mobile in silent place		0.55	
6. Using mobile in library		0.52	
12. Public transport		0.26	
7. Easy to live without mobile			0.87
1. Easy not to use mobile			0.84
10. Lost without mobile			0.74
4. Difficult not to use mobile			0.73
13. Hard to turn off mobile			0.72

Note: PMPUQ-SV: Problematic mobile phone use questionnaire short version.

**Table 4:** Individual confirmatory factor analyses across all samples and for each language of the PMPUQ-SV 3.2. Measurement invariance.

Version	*n*	df	χ2	*p*	CFI	TLI	RMSEA	RMSEA 90% CI	pClose	SRMR
All languages	3038	87	1858.371	<0.001	0.947	0.936	0.082	0.079–0.085	<0.000	0.074
German	383	87	231.642	<0.001	0.972	0.966	0.066	0.056–0.076	<0.000	0.069
French	1076	87	589.755	<0.001	0.973	0.968	0.073	0.068–0.079	<0.000	0.075
English	117	87	108.124	=0.062	0.976	0.972	0.046	0.000–0.072	<0.000	0.106
Finnish	449	87	383.929	<0.001	0.924	0.908	0.087	0.078–0.096	<0.000	0.092
Spanish	156	87	186.553	<0.001	0.940	0.928	0.086	0.069–0.103	=0.001	0.115
Italian	288	87	337.184	<0.001	0.915	0.897	0.100	0.089–0.111	<0.000	0.108
Polish	258	87	303.358	<0.001	0.880	0.856	0.098	0.086–0.111	<0.000	0.112
Hungarian	311	87	236.557	<0.001	0.954	0.945	0.074	0.063–0.086	<0.000	0.103

Note: PMPUQ-SV = Problematic mobile phone use questionnaire short version; χ^2^ = Chi-square value, CFI = comparative fit index, TLI = Tucker–Lewis index, RMSEA = root mean squared error of approximation, pClose = provides a one-sided test of the null hypothesis that the RMSEA is equal to 0.05 in the population, SRMR = standardized root mean square residual.

**Table 5:** Measurement invariance procedure conducted between German, French, Finnish, Spanish, and Hungarian for PMPUQ-SV.

Invariance	df	χ^2^	*p*	CFI	TLI	RMSEA	RMSEA 90% CI	pClose	SRMR	Δχ^2^	Δdf	ΔRMSEA	ΔCFI	ΔSRMR
Configural	435	1567	<0.001	0.964	0.956	0.074	0.070–0.078	<0.001	0.084					
Config vs. metric ***										133	24	0.002	0.001	0.009
Metric	483	1654	<0.001	0.962	0.959	0.072	0.068–0.075	<0.001	0.093					
Metric vs. Scalar ***										165	54	0.003	0.012	0.006
Scalar	591	2154	<0.001	0.950	0.955	0.075	0.071–0.078	<0.001	0.087					
Scalar vs. Strict ***										272	30	0.005	0.014	0.008
Strict	651	2643	<0.001	0.936	0.948	0.080	0.077–0.084	<0.001	0.095					
Strict vs. Means ***										345	6	0.007	0.012	0.002
Means	663	3038	<0.001	0.924	0.939	0.087	0.084–0.090	<0.001	0.097					

Note: PMPUQ-SV: Problematic mobile phone use questionnaire short version; Satorra-Bentler Δχ^2^ Tests: Config vs. Metric: X^2^(5) = 23.930, *p* < 0.001, Metric vs Scalar: X^2^(11) = 29.202, *p* < 0.001, Scalar vs Strict: X^2^(7) = 69.105, *p* < 0.001, Strict vs Means: X^2^(1) = 28.590, *** *p* < 0.001.

**Table 6:** Item response rates per response category across all languages for PMPUQ-SV.

		All Languages			
Factor	Item	Strongly Agree (%)	Agree (%)	Disagree (%)	Strongly Disagree (%)
Dangerous	14. Driving concentration ^R^	1.8	3.1	13.1	82.0
	11. Driving danger mobile ^R^	4.2	4.5	16.1	75.1
	2. Use mobile when driving ^R^	4.7	11.4	20.0	63.9
	8. Dangerous situations ^R^	8.1	12.3	23.3	56.3
	5. Avoid using on motorway	77.3	10.2	6.1	6.4
Prohibited	9. Use mobile where forbidden ^R^	3.4	10.2	27.7	58.8
	3. Don’t use when forbidden	55.1	22.2	16.7	6.0
	15. Use mobile in silent place	63.0	27.2	6.9	2.9
	6. Using mobile in library	27.7	23.7	34.0	14.6
	12. Public transport	64.1	28.3	5.0	2.6
Dependent	7. Easy to live without mobile	21.4	25.6	37.5	15.6
	1. Easy not to use mobile	26.6	26.1	31.9	15.3
	10. Lost without mobile ^R^	11.2	27.3	32.3	29.2
	4. Difficult not to use mobile ^R^	9.7	28.0	32.8	29.5
	13. Hard to turn off mobile ^R^	9.4	19.8	32.1	38.7
	Mean *	*51.2*	*24.0*	*17.0*	*7.7*

Note: PMPUQ-SV: Problematic mobile phone use questionnaire short version; ^R^ = reverse coded for questionnaire validations, but actual score given here. * = mean when items 14, 11, 2, 8, 9, 10, 4, and 13 are reversed.

## Appendix A

**English**

In relation with your mobile phone/smartphone, please answer these questions on a scale from 1 to 4, the numbers corresponding to: 1 “Strongly agree”, 2 “Agree”, 3 “Disagree”, 4 “Strongly disagree”

The statement suits you:

1. It is easy for me to spend all day not using my mobile phone.
2. I use my mobile phone while driving.
3. I don’t use my mobile phone when it is completely forbidden to use it.
4. Is it hard for me not to use my mobile phone when I feel like it.
5. I try to avoid using my mobile phone when driving on the motorway.
6. I don’t use my mobile phone in a library.
7. I can easily live without my mobile phone.
8. I use my mobile phone in situations that would qualify as dangerous.
9. I use my mobile phone where it is forbidden to do so.
10. I feel lost without my mobile phone.
11. While driving, I find myself in dangerous situations because of my mobile phone use.
12. When using my mobile phone on public transport, I try not to talk too loud.
13. It is hard for me to turn my mobile phone off.
14. I use my mobile phone while driving, even in situations that require a lot of concentration.
15. I try to avoid using mobile phone where people need silence.

**French**

Concernant votre téléphone portable/smartphone, veuillez répondre à ces questions selon une échelle allant de 1 à 4, ces chiffres correspondant à : 1 “Tout à fait “, 2 “Plutôt bien “, 3 “Plutôt mal“, 4“Pas du tout “

L’énoncé vous correspond:

1. Il est facile pour moi de passer toute une journée sans utiliser mon téléphone portable.
2. Je téléphone en conduisant.
3. Je n’utilise pas mon téléphone portable dans des lieux où il est formellement interdit de le faire.
4. Il m’est difficile de ne pas utiliser mon téléphone portable lorsque j’en ai envie.
5. J’évite d’utiliser mon téléphone portable quand je conduis sur l’autoroute.
6. Je n’utilise pas mon téléphone portable quand je suis dans une bibliothèque.
7. Je peux facilement me passer de mon téléphone portable.
8. Je me sers de mon téléphone portable dans des situations que je peux qualifier de «dangereuses».
9. Je me sers de mon téléphone portable dans des lieux où la loi l’interdit.
10. Je me sens perdu quand je n’ai pas mon téléphone portable.
11. En conduisant, je me retrouve en situation délicate alors que j’utilise mon téléphone portable.
12. Quand je téléphone dans les transports publics, je fais attention à ne pas parler trop fort.
13. Il est pénible pour moi d’éteindre mon téléphone portable.
14. En conduisant, j’utilise mon téléphone portable dans des situations qui demandent une concentration importante.
15. J’évite d’utiliser mon téléphone portable dans des endroits où il faut être silencieux.

**German**

Bitte beantworten Sie die nächsten Fragen in Bezug auf Ihr Smartphone/Handy auf einer Skala von 1 bis 4, die Zahlen entsprechend: 1 “Stimme sehr zu”, 2 “Stimme zu”, 3 “Stimme nicht zu”, 4 “ Stimme überhaupt nicht zu”

Wählen Sie die Aussage, die am besten zu Ihnen passt:

1. Es fällt mir leicht, den Tag zu verbringen, ohne mein Handy zu benutzen.
2. Ich benutze mein Handy, während ich Auto fahre.
3. Ich gebrauche mein Handy nicht, wenn es absolut verboten ist es zu benutzen.
4. Es fällt mir schwer mein Handy nicht zu verwenden, wenn mir danach ist.
5. Ich versuche zu vermeiden, mein Handy während der Autobahnfahrt zu benutzen.
6. Ich benutze mein Handy nicht in einer Bibliothek.
7. Ohne mein Handy kann ich problemlos leben.
8. Ich benutze mein Handy in Situationen, die als gefährlich gelten würden.
9. Ich verwende mein Handy an Orten, an denen die Nutzung verboten ist.
10. Ohne mein Handy fühle ich mich verloren.
11. Während ich Auto fahre, bringe ich mich in gefährliche Situationen, weil ich gleichzeitig mein Handy benutze.
12. Bei der Verwendung meines Mobiltelefons in öffentlichen Verkehrsmitteln versuche ich nicht zu laut zu sprechen.
13. Es fällt mir schwer, mein Handy auszuschalten.
14. Ich benutze mein Handy während der Autofahrt, selbst in Situationen, die viel Aufmerksamkeit erfordern.
15. Ich versuche meine Handynutzung in Situationen zu vermeiden, in denen Menschen Ruhe brauchen.

**Hungarian**

Kérjük, válaszold meg az alábbi kérdéseket a mobiltelefonod használatával kapcsolatban egy egytől négyig terjedő skálán, ahol: 1 “Teljesen egyetértek”, 2 “Egyetértek”, 3 “Nem értek egyet”, 4 “Egyáltalán nem értek egyet”

Mennyire értesz egyet az alábbi állításokkal?

1. Könnyű számomra egy egész napot eltölteni anélkül, hogy használnám a mobilomat.
2. Használom a mobilomat vezetés közben.
3. Nem használom a mobilomat olyankor, amikor ez egyértelműen tilos.
4. Nehezemre esik, hogy ne használjam a mobilomat bármikor, amikor csak kedvem van hozzá.
5. Igyekszem nem használni a mobilomat, amikor autópályán vezetek.
6. Nem használom a mobilomat, ha könyvtárban vagyok.
7. Jól elvagyok a mobilom nélkül.
8. Olyan helyzetekben is használom a mobilomat, amikor az mások szerint veszélyes lehet.
9. Olyan helyen is használom a mobilomat, ahol ez tilos.
10. Elveszettnek érzem magam a mobilom nélkül.
11. Sokszor veszélyes helyzetekben találom magam vezetés közben a mobilhasználatom miatt.
12. Amikor tömegközlekedési eszközön használom a mobilomat, igyekszem nem túl hangosan beszélni.
13. Nehezemre esik kikapcsolni a mobilomat.
14. Még olyan helyzetekben is használom a mobilomat vezetés közben, amik nagy koncentrációt igényelnek.
15. Igyekszem nem használni a mobilomat olyan helyeken, ahol másoknak csendre van szükségük.

**Finnish**

Alla väittämiä matka-/älypuhelimenne käytöstä. Vastatkaa seuraaviin väittämiin valitsemalla itseänne parhaiten kuvaava vaihtoehto asteikolla 1–4: 1 ”Täysin samaa mieltä”, 2 ”Jokseenkin samaa mieltä”, 3 ”Jokseenkin eri mieltä”, 4 ”Täysin eri mieltä”

1. Minulle on helppoa olla koko päivä käyttämättä matkapuhelintani.
2. Käytän matkapuhelinta autolla ajaessani.
3. En käytä matkapuhelintani silloin, kun sen käyttö on ehdottomasti kielletty.
4. On vaikeaa olla käyttämättä matkapuhelinta silloin kun haluaisin käyttää sitä.
5. Yritän välttää matkapuhelimen käyttöä moottoritiellä ajaessani.
6. En käytä matkapuhelinta kirjastossa.
7. Voin helposti elää ilman matkapuhelinta.
8. Käytän matkapuhelintani tilanteissa, jotka voidaan luokitella vaarallisiksi.
9. Käytän matkapuhelintani silloinkin, kun sen käyttö on kielletty.
10. Tunnen olevani hukassa ilman matkapuhelintani.
11. Ajaessani huomaan olevani vaarallisissa tilanteissa matkapuhelimen käyttöni seurauksena.
12. Käyttäessäni matkapuhelinta joukkoliikennevälineissä yritän olla puhumatta kovalla äänellä.
13. Minulle tuottaa vaikeuksia laittaa matkapuhelimeni pois päältä.
14. Käytän matkapuhelinta ajaessani autolla myös tilanteissa, jotka vaativat erityistä tarkkaavaisuutta.
15. Yritän välttää matkapuhelimen käyttöä tilanteissa, joissa tarvitaan hiljaisuutta.

**Italian**

Per favore, indica quanto ciascuna affermazione è adatta a descrivere l’uso che fai del tuo cellulare/ smartphone, su una scala da 1 a 4. I numeri corrispondono a: 1. “Fortemente d’accordo”, 2. “D’accordo”, 3. “In disaccordo”, 4. “Fortemente in disaccordo”

1. E’ facile per me trascorrere tutto il giorno senza utilizzare il cellulare.
2. Utilizzo il cellulare mentre guido.
3. Non uso il cellulare quando è assolutamente proibito usarlo.
4. E’ difficile per me non utilizzare il cellulare quando mi sento di farlo.
5. Cerco di evitare di utilizzare il cellulare quando guido in autostrada.
6. Non uso il cellular in biblioteca.
7. Posso vivere facilmente senza il cellulare.
8. Uso il cellulare in situazioni che potrebbero essere considerate pericolose.
9. Uso il cellulare dove è proibito farlo.
10. Mi sento perso senza il cellulare.
11. Quando guido mi ritrovo in situazioni pericolose perchè utilizzo il cellulare.
12. Quando uso il cellulare su un mezzo di traporto pubblico, cerco di non parlare troppo forte.
13. E’ difficile per me spegnere il cellulare.
14. Uso il cellulare quando guido, anche in situazioni che richiedono molta concentrazione.
15. Cerco di evitare di usare il cellulare nei posti in cui la gente ha bisogno di silenzio.

**Spanish**

En relación con su móvil/smartphone, responda por favor a estas cuestiones en una escala de valoración del 1 al 4, los números corresponden a: 1 “Muy de acuerdo”, 2 “De acuerdo”, 3 “En desacuerdo”, 4 “Muy en desacuerdo”

La declaración que más le convenga:

1. Es fácil para mí pasar todo el día sin usar el móvil.
2. Uso el móvil mientras conduzco.
3. Yo no uso el móvil cuando está completamente prohibido.
4. Es difícil para mí no usar el móvil cuando quiero usarlo.
5. Trato de evitar el uso del móvil cuando conduzco por la autopista.
6. No uso el móvil en una biblioteca.
7. Puedo vivir fácilmente sin mi móvil.
8. Uso el móvil en situaciones que podrían considerarse peligrosas.
9. Uso el móvil donde está prohibido utilizarlo.
10. Me siento perdido sin el móvil.
11. Me he encontrado en situaciones peligrosas debido al uso del móvil mientras conducía.
12. Cuando uso el móvil en el transporte público, trato de no hablar demasiado alto.
13. Es difícil para mí apagar el móvil.
14. Uso el móvil durante la conducción, incluso en situaciones que requieren mucha concentración.
15. Trato de evitar el uso del móvil en lugares en que la gente necesita silencio (o se ruega silencio).

**Polish**

Proszę odpowiedzieć na pytania dotyczące Pana(i) telefonu/smartfona, posługując się skalą, w której kolejne cyfry od 1 do 4 znaczą: 1 “Całkowicie“, 2 “Raczej tak“, 3 “Raczej nie“, 4 “Wcale“

Stwierdzenie odpowiadające Panu(i):

1. Łatwo mi spędzić cały dzień bez używania mojego telefonu komórkowego.
2. Rozmawiam przez telefon prowadząc samochód.
3. Nie używam telefonu w miejscach, w których formalnie jest to zakazane.
4. Trudno mi się powstrzymać od używania mojego telefonu komórkowego, kiedy mam na to ochotę.
5. Staram się nie używać telefonu, kiedy prowadzę saochód na autostradzie.
6. Nie używam telefonu kiedy jestem w bibliotece.
7. Z łatwością mogę obejść się bez mojego telefonu.
8. Używam mojego telefonu w sytuacjach, które uważam za « niebezpieczne ».
9. Używam mojego telefonu w miejscach, w których prawo tego zakazuje.
10. Czuję się zagubiony, gdy nie mam ze sobą mojego telefonu.
11. Prowadząc, zdarza mi się znaleźć w trudnej sytuacji podczas gdy używam telefonu komórkowego.
12. Kiedy rozmawiam przez telefon w środkach komunikacji publicznej, zwracam uwagę by nie rozmawiać zbyt głośno.
13. Źle się czuję, kiedy wyłączam mój telefon, nie lubię tego.
14. Prowadząc samochód używam telefonu w sytuacjach, które wymagają szczególnej koncentracji.
15. Unikam używania telefonu w miejscach, w których należy zachować ciszę.
